# Negative and disorganized symptoms mediate the relationship between verbal learning and global functioning in adolescents with early-onset psychosis

**DOI:** 10.1007/s00787-020-01479-7

**Published:** 2020-02-08

**Authors:** Runar Elle Smelror, Bjørn Rishovd Rund, Vera Lonning, Kjetil Nordbø Jørgensen, Kirsten Wedervang-Resell, Ole A. Andreassen, Torill Ueland, Anne M. Myhre, Ingrid Agartz

**Affiliations:** 1 NORMENT, Institute of Clinical Medicine, University of Oslo, Oslo, Norway; 2 Department of Psychiatric Research, Diakonhjemmet Hospital, Oslo, Norway; 3 Department of Psychology, University of Oslo, Oslo, Norway; 4 Research Department, Vestre Viken Hospital Trust, Drammen, Norway; 5 Division of Mental Health and Addiction, Oslo University Hospital, Oslo, Norway; 6 Department of Clinical Neuroscience, Centre for Psychiatric Research, Karolinska Institutet, Stockholm, Sweden

**Keywords:** MATRICS, MCCB, Early-onset schizophrenia, Neurocognition

## Abstract

Neurocognitive deficits are associated with impaired global functioning and psychotic symptoms. However, whether symptoms can mediate the relationship between neurocognition and global functioning in adolescent psychosis is unclear. Here, we investigated if symptoms assessed with the Positive And Negative Syndrome Scale (PANSS), mediated the relationship between neurocognitive performance and global functioning in adolescents with non-affective early-onset psychotic disorders (EOP). Sixty-one adolescent EOP patients (age 12–18 years) from 2 Norwegian clinical cohorts were included. Linear regression models were applied to investigate associations between neurocognitive domains from the MATRICS Consensus Cognitive Battery (MCCB) and global functioning. PANSS symptoms were analyzed using the Wallwork/Fortgang five-factor model. Using the INDIRECT macro for SPSS, mediation effects were tested using bootstrapping with 95% bias corrected confidence intervals. Verbal learning was positively associated with global functioning (*P* < 0.001) and negatively associated with the disorganized symptom factor (*P* = 0.002), controlling for age, sex and cohort. Testing of indirect effects, controlling for age, sex and cohort, showed that the Negative (point estimate = 1.56, 95% CI 0.22, 3.47) and Disorganized (point estimate = 1.24, 95% CI 0.05, 3.69) symptom factors significantly mediated the relationship between verbal learning and global functioning. We found that verbal learning, negative and disorganized symptoms influenced global functioning in adolescents with EOP, while reality-distorted positive symptoms did not. These results suggest that assessing these domains in EOP is helpful for planning treatment and rehabilitation programs focusing on functional outcome.

**Electronic supplementary material:**

The online version of this article (10.1007/s00787-020-01479-7) contains supplementary material, which is available to authorized users.

## Introduction

Early-onset psychosis (EOP) includes psychotic disorders with age of onset before 18 years. Early-onset schizophrenia (EOS) is one of the leading causes of disease burden in adolescents between 15–19 years [1]. It is widely regarded as a neurodevelopmental disorder [2–7] with neurocognitive deficits as a core feature of the illness [8]. Neurocognitive deficits are present before the onset of illness [9–12], relatively stable over time [13–16] and found in children with familial high risk of schizophrenia [17]. Although the neurocognitive deficits in EOP are similar to adult schizophrenia patients [18, 19], the deficits in adolescents seem to be greater than in adults [8, 20, 21]. These findings suggest that abnormal neurodevelopment in schizophrenia is present early in life in a period of extensive brain maturation and before the onset of illness [22]. EOP is thus important to investigate as adolescents are less influenced by any secondary effects of the disease, such as long-term medical treatment, unhealthy lifestyle, etc. Increased knowledge of cognitive functioning and neurodevelopment during this life period can provide new insight into the mechanisms contributing to impaired functional outcome and the development of psychosis [5, 6].

Long-term follow-up studies have showed that 42–74% of individuals with EOP had severely impaired functional outcome after 9–42 years (e.g. lived in a supervised home/institution, had symptoms most/all the time, had reduced/no work) [19, 23–28]. Few studies have investigated putative associations between neurocognition and functional outcome in EOP. Two studies, including patients with EOS, found that neurocognitive deficits in speed of processing, verbal learning, attention, working memory and executive functioning were associated with impaired functional outcome at follow-up [19, 29]. As neurocognition is not part of the diagnostic criteria of psychotic disorders, symptom reduction on clinical rating scales (e.g. the Positive and Negative Syndrome Scale (PANSS) [30]) is often used as the only indicator of treatment success in clinical trials of schizophrenia. While the original PANSS includes three subscales, factor analyses show that five-factor models show better statistical validity, with the Wallwork/Fortgang model [31] showing the most optimal statistical fit for adult first-episode psychosis patients [32]. This model includes Positive, Negative, Disorganized/concrete, Excited and Depressed symptom factors, and separates reality-distorted positive symptoms (e.g. delusions and hallucinations) from disorganized symptoms (e.g. conceptual disorganization and reduced attention). This is in line with factor analyses suggesting that disorganized symptoms represent a different construct than positive symptoms [33, 34]. This is important as negative and disorganized symptoms have been more strongly associated with neurocognitive deficits than positive symptoms [35]. Stronger associations have also been found between negative symptoms and impaired functional outcome in EOP [36] and adult schizophrenia [37, 38], compared to positive symptoms. A recent study of adult first-episode psychosis patients and a former meta-analysis found that negative symptoms partially mediated the relationship between neurocognitive performance and global functioning in adult patients, while no significant association was found for reality-distorted positive symptoms [39, 40]. Disorganized symptoms were not assessed in these studies.

To the best of our knowledge, no studies have investigated if characteristic symptoms of psychosis mediate the relationship between neurocognitive performance and global functioning in adolescents with EOP. This may shed light on the nature of this relationship and help identify which psychotic symptoms are relevant predictors of functioning in adolescent psychosis. The present study examined whether neurocognitive performance, assessed with the MCCB, was associated with global functioning in EOP, and whether symptom domains from the Wallwork/Fortgang five-factor model mediated this relationship. Based on previous research in adolescent schizophrenia [19, 29], we hypothesized that speed of processing, verbal learning, attention, working memory and global cognition would be associated with global functioning in EOP. Furthermore, based on a previous systematic review [35] and research in adult schizophrenia [39, 40], we hypothesized that the Negative and Disorganized/concrete symptom factors would mediate this relationship, while no significant mediation effects would be found for positive symptoms.

## Materials and methods

### Participants

A total of 61 adolescents with early-onset non-affective psychotic disorders (EOP) were combined from 2 Norwegian clinical cohorts at the University of Oslo (the Thematically Organized Psychosis Study for Youth [Youth-TOP] and the Early-Onset Study). All participants were recruited from adolescent psychiatric inpatient units and outpatient clinics in the Oslo region from 2013–2016 (Youth-TOP) and 2005–2007 (Early-Onset Study). The Youth-TOP has an ongoing inclusion of participants and no neurocognitive patient data have previously been reported (see [41] for more information about patient characterization). Neurocognitive data from the Early-Onset Study have been published in previous papers, e.g. [15, 18, 42]. The inclusion criteria in both cohorts were: (1) Non-affective early-onset psychosis (schizophrenia, schizoaffective disorder, schizophreniform disorder, psychotic disorder not otherwise specified, brief psychotic disorder), according to the *Diagnostic and Statistical Manual of Mental Disorders, fourth edition* (DSM-IV), (2) age between 12 and 18 years, (3) written informed consent obtained from participants, parents or guardians (if the participant was under 16 years), (4) language abilities to complete the interviews and self-rating questionnaires. The exclusion criteria in both cohorts were: (1) IQ below 70, (2) previous moderate/severe head injury, (3) a diagnosis of substance-induced psychosis, (4) organic brain disease. Demographic and clinical information of the participants are provided in Table 1.

**Table 1:** Demographic and clinical characteristics of the participants, and statistical tests for differences between the two cohorts

	Youth-TOP	Early-Onset Study	Test statistics
	*N* = 34	*N* = 27	
Inclusion years	2013–2016	2005–2007	
Diagnostic assessment	K-SADS-PL^a^	SCID-I^b^	
CGAS/GAF-F^c^ (SD)	43.2 (10.3)	48.0 (15.2)	*t*(44) = 1.41, *P* = 0.17
Sex, female (%)	21 (62)	14 (52)	*χ*^2^(1) = 0.61, *P* = 0.44
Hand dominance, right (%)	31 (91)	23 (85)	*χ*^2^(1) = 0.53, *P* = 0.47
Mother’s education, years (SD)	14.7 (2.7)	12.9 (2.8)	***t*(56) = − 2.51, *P* = 0.02**
Age, years (SD)	16.2 (1.3)	15.9 (1.8)	*t*(59) = − 0.13, *P* = 0.53
IQ (SD)	100.4 (12.4)	95.2 (14.4)	*t*(59) = − 1.51, *P* = 0.14
Ethnicity, caucasian (%)	30 (88)	21 (78)	*χ*^2^(1) = 1.20, *P* = 0.27
Diagnosis (%)			*χ*^2^(4) = 3.75, *P* = 0.44
Schizophrenia	21 (62)	12 (44)	
Schizoaffective disorder	1 (3)	3 (11)	
Schizophreniform disorder	0 (0)	1 (4)	
Brief psychotic disorder	1 (3)	1 (4)	
Psychosis not otherwise specified	11 (32)	10 (37)	
Daily nicotine use, yes (%)	9 (27)	6 (23)	*χ*^2^(1) = 0.09, *P* = 0.76
Alcohol use, yes (%)	12 (35)	17 (63)	**χ**^**2**^**(1) = 4.62, *P* = 0.03**
Cannabis use, yes (%)	8 (24)	4 (15)	*χ*^2^(1) = 0.72, *P* = 0.40
Antipsychotic med., yes, *n* (%)	18 (53)	19 (70)	*χ*^2^(1) = 1.92, *P* = 0.17
Aripiprazole	9 (50)	2 (11)	
Risperidone	3 (16)	2 (11)	
Quetiapine	4 (22)	6 (31)	
Olanzapine	1 (6)	6 (31)	
Ziprasidone	0 (0)	2 (11)	
Clozapine	1 (6)	1 (5)	
Age of onset, mean years (SD)	14.3 (1.9)	14.1 (2.0)	*t*(59) = − 0.41, *P* = 0.68
DUP^d^, weeks (SD)	38.5 (47.6)	35.0 (51.5)	*t*(59) = 0.28, *P* = 0.78
PANSS^e^ (SD)			
Positive	17.3 (4.1)	14.9 (4.2)	***t*(59) = − 2.26, *P* = 0.03**
Negative	19.2 (8.0)	12.2 (5.3)	***t*(57) = − 4.10, *P* < 0.001**
General	36.5 (8.1)	29.7 (7.4)	***t*(59) = − 3.37, *P* = 0.001**
PANSS Wallwork/Fortgang (SD)			
Positive	2.9 (0.89)	2.5 (0.83)	***t*(59) = − 1.99, *P* = 0.05**
Negative	2.8 (1.18)	1.9 (0.76)	***t*(57) = − 3.45, *P* = 0.001**
Disorganized/concrete	2.2 (0.94)	1.6 (0.64)	***t*(58) = − 3.02, *P* = 0.004**
Excited	1.7 (0.55)	1.7 (0.63)	*t*(59) = − 0.04, *P* = 0.97
Depressed	2.8 (0.88)	2.6 (1.13)	*t*(49) = − 0.87, *P* = 0.39

^a^Schedule for Affective Disorders and Schizophrenia for School Aged Children-Present and Lifetime Version (K-SADS-PL [43])

^b^Structured Clinical Instrument of Diagnosis for DSM-IV Axis I Disorders, modules A-D (First et al. [44])

^c^Children’s Global Assessment Scale (CGAS) [45]. Global Assessment of Functioning (GAF) Scale (split DSM-IV version) [46]

^d^Duration of untreated psychosis

^e^Positive And Negative Syndrome Scale [30]

Bold values indicate *P* ≤ 0.05

### Clinical measures

Diagnoses were confirmed using the Schedule for Affective Disorders and Schizophrenia for School Aged Children-Present and Lifetime Version (K-SADS-PL) [43] in the Youth-TOP study and the Structural Clinical Instrument of Diagnosis for DSM-IV Axis I disorders (SCID-I), modules A-D [44], in the Early-Onset Study. For both cohorts, current psychopathology was assessed using the PANSS [30], and analyzed using the Wallwork/Fortgang five-factor model, consisting of Positive, Negative, Disorganized/concrete, Excited, and Depressed symptom factors [31]. Although the Wallwork/Fortgang model has not been validated in adolescents, it has shown the most optimal fit for adult first-episode schizophrenia patients, aged 18–65 years [32].

The interviewers in both studies were clinical psychologists or medical doctors who had completed an inter-rater reliability training course (≥ 80% inter-rater reliability) in PANSS assessment based on a training program developed at the University of California, Los Angeles. All interviewers participated in regular diagnostic consensus meetings led by a senior clinical researcher in the field of psychosis.

### Global functioning

The participants’ level of global functioning was assessed using the Children’s Global Assessment Scale (CGAS) [45] in the Youth-TOP study and the Global Assessment of Functioning Scale, split DSM-IV version (GAF-F) [46, 47], in the Early-Onset Study. Both rating scales are based on the Global Assessment Scale [48], so the scoring of functioning is similar, ranging from 0 (lowest) to 100 (highest). The scales include behavior and activities indicative of daily functioning, such as social functioning, school performance and independent living [39, 49]. Despite minor differences in the naming of anchor points (e.g. scores from 51–60 are defined as “variable functioning” in the CGAS and “moderate functioning” in the GAF-F), the descriptions of functioning within each anchor point is similar between the scales (see [50] for more information about the scales). Although the anchor points in the CGAS contain both functioning and symptoms, while the GAF-F only contains functioning, we considered the two scales to be adequately comparable to allow us to merge for analysis of global functioning in the combined cohort. No significant differences were found between the CGAS and the GAF-F scores between the two cohorts (Table 1).

### Neurocognitive measures

The participants underwent intelligence assessment using the Wechsler Abbreviated Scale of Intelligence [51]. Neurocognitive profiling was performed using a licensed translated version of the MATRICS Consensus Cognitive Battery (MCCB) [52], with the exception of the social cognition test Mayer–Salovey–Caruso Emotional Intelligence Test (MSCEIT) [53]. The MCCB was developed for clinical trials of schizophrenia [54] and has been successfully used to assess cognitive functioning in children [55] and adolescents [18, 56]. The nine included tests covered six neurocognitive domains: (1) *Speed of processing*, measured with the BACS Symbol coding [57], Trail making test, part A (TMT-A) [58], and Category fluency: Animal naming [59], (2) *Attention/vigilance*, measured with the Continuous performance test, identical pairs (CPT-IP) [60], (3) *Working memory*, measured with the WMS-III Spatial span [61] and Letter-number span [62], (4) *Verbal learning*, measured with the Hopkins verbal learning test, revised (HVLT-R) [63], (5) *Visual learning*, measured with the Brief visuospatial memory test, revised (BVMT-R) [64], and (6) *Reasoning and problem solving*, measured with the NAB Mazes [65]. The HVLT-R test was originally validated for age ≥ 16 years but has been successfully used and standardized in children and adolescents [56, 66-68].

### Statistical analyses

Statistical analyses were performed using IBM SPSS Statistics for Windows, Version 25. All tests were two-tailed. Independent-sample *t* tests were used for analyses of cohort comparisons of continuous variables and chi-square (*χ*^2^) tests for comparisons of categorical data. We transformed the neurocognitive raw test scores from the MCCB in the total sample to standard scores (*z* scores) using the SPSS standardization function. High scores on the TMT-A test indicate impairment and were reversed. In domains containing more than one subtest and for the global cognition score, composite scores were calculated by summating the standard scores of relevant tests and transforming the sum scores to standard scores using the same procedure as described above. This procedure is in line with previous MCCB standardization studies [56, 69]. In the main analysis examining associations between neurocognition and global functioning, we applied separate linear regression models using the neurocognitive domains and global cognition as predictor variables and global functioning as the outcome variable. IQ was not included as it has been found to be moderately to highly correlated with the MCCB domains and the global cognition score [70, 71]. Age, sex and cohort were added as covariates as age and sex effects have been found in adolescent performance on the MCCB [56] and to avoid potential systematic cohort differences. Correction for multiple testing was performed using Bonferroni, in which *P* ≤ 0.007 was considered significant in the main analysis (*P* = 0.05/7 neurocognitive domains). Based on the results from the main analysis, we examined associations between (1) neurocognition and symptoms, using the mean symptom factor scores as predictor variables and the relevant neurocognitive domains as outcome variables, controlling for age, sex and cohort; and (2) symptoms and global functioning, using the symptom factors as predictor variables and global functioning as the outcome variable, controlling for age, sex and cohort. In both secondary analyses, *P* ≤ 0.01 was considered significant (*P* = 0.05/5 symptom factors). Lastly, to examine if symptoms mediated the relationship between the relevant neurocognitive domains and global functioning, we applied separate mediation analyses of the five symptom factors, controlling for age, sex and cohort, using the INDIRECT macro for SPSS [72]. The indirect effects were tested using 95% bias corrected (BC) bootstrap intervals with 5000 bootstrap samples. The indirect effects were considered statistically significant if the confidence interval of each point estimate did not include zero (null value). Bootstrapping was preferred over the product-of-coefficients approach (i.e. the Sobel test), because no assumptions of normal distributions are required [73] and because this approach is considered to be more valid for testing of indirect effects [74].

## Results

### Patient characteristics

Demographic and clinical characteristics of the two cohorts are provided in Table 1. The participants in the Youth-TOP study had significantly higher PANSS scores (Positive: *t*(59) = − 2.26, *P* = 0.03; Negative: *t*(57) = − 4.10, *P* < 0.001; General: *t*(59) = − 3.37, *P* = 0.001) and significantly higher scores on the Wallwork/Fortgang Positive [*t*(59) = − 1.99, *P* = 0.05], Negative [*t*(57) = − 3.45, *P* = 0.001] and Disorganized/concrete [*t*(58) = − 3.02, *P* = 0.004] symptom factors, compared to the participants in the Early-Onset Study. Furthermore, the participants in the Youth-TOP study had mothers with significantly more years of education than the participants in the Early-Onset Study [*t*(56) = − 2.51, *P* = 0.02], while a significantly higher proportion of participants in the Early-Onset Study had used alcohol compared to the participants in the Youth-TOP study [*χ*^2^ (1) = 4.62, *P* = 0.03]. When comparing neurocognitive performance between the two cohorts, the participants in the Youth-TOP study had significantly higher scores on the Spatial span test [*t*(58) = − 2.59, *P* = 0.01), Working memory domain [*t*(58) = − 2.55, *P* = 0.01] and on the Global cognition score [*t*(50) = − 2.73, *P* = 0.01] compared to the patients in the Early-Onset Study. MCCB test and domain scores are presented in Table 2.

**Table 2:** MCCB test and domain scores of the participants, and statistical tests for differences between the two cohorts

MCCB test and domain scores	Youth-TOP	Early-Onset Study	Test statistics
	*N* = 34	*N* = 27	
Speed of processing, *Z* score	0.15 (0.98)	− 0.19 (1.01)	*t*(57) = − 1.30, *P* = 0.20
BACS symbol coding	49.61 (12.98)	46.59 (11.26)	*t*(58) = − 0.95, *P* = 0.35
Animal naming	21.09 (5.44)	18.35 (5.46)	*t*(58) = − 1.93, *P* = 0.06
Trail making test A	36.22 (12.74)	35.92 (14.61)	*t*(58) = − 0.08, *P* = 0.93
Attention/vigilance: CPT-IP d´	1.77 (0.68)	1.70 (0.70)	*t*(54) = − 0.38, *P* = 0.71
Working memory, *Z* score	0.28 (0.96)	− 0.36 (0.95)	***t*(58) = − 2.55, *P* = 0.01**
WMS-III spatial span	16.62 (2.67)	14.81 (2.70)	***t*(58) = − 2.59, *P* = 0.01**
Letter-number span	12.94 (3.14)	11.70 (2.32)	*t*(59) = − 1.71, *P* = 0.09
Verbal learning: HVLT-R	25.52 (5.19)	22.81 (5.50)	*t*(58) = − 1.95, *P* = 0.06
Visual learning: BVMT-R	25.38 (6.79)	22.93 (8.78)	*t*(48) = − 1.20, *P* = 0.24
Reasoning and problem-solving: NAB Mazes	20.29 (4.45)	17.85 (5.69)	*t*(59) = − 1.88, *P* = 0.07
Global cognition, *Z* score	− 0.34 (0.90)	− 0.37 (0.98)	***t*(50) = − 2.73, *P* = 0.01**

Bold values indicate *P* ≤ 0.05

### Associations between neurocognitive performance, global functioning, and symptoms

**Fig. 1:**
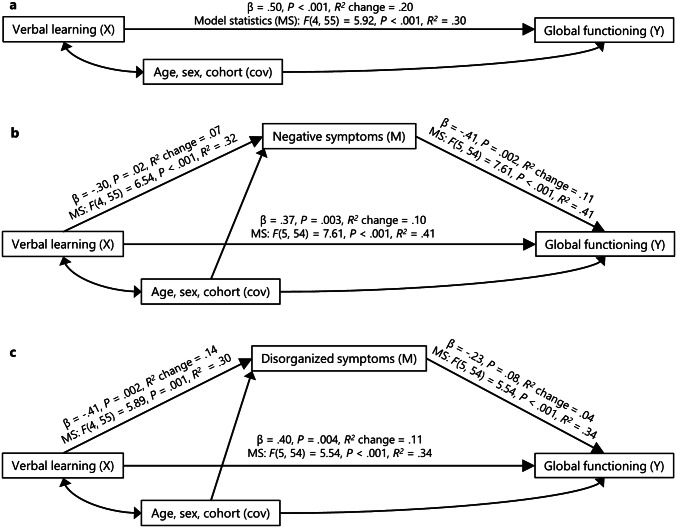
**a** The effect of verbal learning (*X*) on global functioning (*Y*), controlling for age, sex and cohort (*cov*). **b**,** c** Mediation models illustrating that negative and disorganized symptom factors (*M*) significantly mediate the relationship between verbal learning (*X*) and global functioning (*Y*), controlling for age, sex and cohort (*cov*). For more information about the theory and path definitions, see [72]. *MS* model statistics

Verbal learning was the only neurocognitive domain significantly associated with global functioning, controlling for age, sex and cohort (*β* = 0.50, *P* < 0.001, *R*^2^ change = 0.20, model statistics (MS): *F*(4, 55) = 5.92, *P* < 0.001, *R*^2^ = 0.30, Fig. 1a), and thus the only domain included in the subsequent analyses. For the other domains and the global cognition score, no significant associations were found (Speed of processing: *β* = 0.22, *P* = 0.10, *R*^2^ change = 0.05, MS: *F*(4, 54) = 2.35, *P* = 0.07, *R*^2^ = 0.15; attention/vigilance: *β* = − 0.02, *P* = 0.87, *R*^2^ change = 0.00, MS: *F*(4, 51) = 1.04, *P* = 0.40, *R*^2^ = 0.08; working memory: *β* = 0.01, *P* = 0.93, *R*^2^ change = 0.00, MS: *F*(4, 55) = 1.70, *P* = 0.16, *R*^2^ = 0.11; visual learning: *β* = 0.13, *P* = 0.31, *R*^2^ change = 0.02, MS: *F*(4, 56) = 1.89, *P* = 0.12, *R*^2^ = 0.12; reasoning and problem solving: *β* = 0.09, *P* = 0.52, *R*^2^ change = 0.01, MS: *F*(4, 56) = 1.71, *P* = 0.16, *R*^2^ = 0.11; global cognition: *β* = 0.18, *P* = 0.25, *R*^2^ change = 0.03, MS: *F*(4, 47) = 1.42, *P* = 0.24, *R*^2^ = 0.11). When performing separate analyses of the two cohorts, verbal learning was the only neurocognitive domain significantly associated with global functioning in the Early-Onset Study after correcting for multiple testing. None of the associations between the neurocognitive domains and global functioning reached statistical significance in the Youth-TOP. The separate results for the two cohorts are presented in the supplemental material.

When investigating associations between the verbal learning domain and symptoms, controlling for age, sex and cohort, verbal learning was significantly negatively associated with the disorganized/concrete (*β* = − 0.40, *P* = 0.002, *R*^2^ change = 0.13, MS: *F*(4, 55) = 6.49, *P* < 0.001, *R*^2^ = 0.32) symptom factor (*P* ≤ 0.01 considered significant after Bonferroni correction). No significant associations were found for the other symptom factors after correcting for multiple testing (positive: *β* = − 0.08, *P* = 0.52, *R*^2^ change = 0.01, MS: *F*(4, 55) = 3.32, *P* = 0.02, *R*^2^ = 0.19; negative: *β* = − 0.32, *P* = 0.02, *R*^*2*^ change = 0.08, MS: *F*(4, 55) = 4.97, *P* = 0.002, *R*^2^ = 0.27; excited: *β* = − 0.11, *P* = 0.36, *R*^2^ change = 0.01, MS: *F*(4, 55) = 3.45, *P* = 0.01, *R*^2^ = 0.20; depressed: *β* = 0.06, *P* = 0.61, *R*^2^ change = 0.00, MS: *F*(4, 55) = 3.27, *P* = 0.02, *R*^2^ = 0.19).

When investigating associations between symptom factors and global functioning, controlling for age, sex and cohort, significant associations were found for the Negative (*β* = − 0.52, *P* < 0.001, *R*^2^ change = 0.20, MS: *F*(4, 56) = 6.13, *P* < 0.001, *R*^2^ = 0.30) and disorganized/concrete (β = − 0.39, *P* = 0.004, *R*^2^ change = 0.13, MS: *F*(4, 56) = 4.14, *P* = 0.005, *R*^2^ = 0.23) symptom factors (*P* ≤ 0.01 considered significant after Bonferroni correction). The other symptom factors did not reach statistical significance after correcting for multiple testing (Positive: *β* = − 0.20, *P* = 0.13, *R*^2^ change = 0.04, MS: *F*(4, 56) = 2.27, *P* = 0.07, *R*^2^ = 0.14; Excited: *β* = − 0.26, *P* = 0.04, *R*^2^ change = 0.07, MS: *F*(4, 56) = 2.83, *P* = 0.03, model *R*^2^ = 0.17; depressed: *β* = − 0.21, *P* = 0.12, *R*^2^ change = 0.04, MS: *F*(4, 56) = 2.30, *P* = 0.07, model *R*^2^ = 0.14).

### Symptoms as mediators of the association between neurocognitive performance and global functioning

Verbal learning was the only neurocognitive domain significantly associated with global functioning and thus the only domain included in the mediation analyses for testing the indirect effects. The Negative (point estimate = 1.56, BC 95% CI 0.22, 3.47) and the Disorganized/concrete (point estimate = 1.24, BC 95% CI 0.05, 3.69) symptom factors significantly mediated the relationship (CI not including the null value) between verbal learning and global functioning, controlling for age, sex and cohort (i.e. testing the indirect effect of *X* on *Y*, through *M*, shown in Fig. 1b, c). For the other symptom factors, the indirect effects did not reach statistical significance (Positive: point estimate = 0.19, BC 95% CI − 0.33, 1.59; excited: point estimate = 0.34, BC 95% CI − 0.25, 1.81; depressed: point estimate = − 0.17, BC 95% CI − 1.54, 0.65). When performing separate mediation analyses of the two cohorts, the Excited symptom factor was the only factor significantly mediating the relationship between verbal learning and global functioning in the Early-Onset Study. None of the symptom factors significantly mediated the relationship between verbal learning and global functioning in the Youth-TOP. The separate results for the two cohorts are presented in the supplemental material.

## Discussion

The purpose of this study was to examine associations between neurocognitive performance and global functioning, and to test whether symptoms mediated this relationship in adolescents with EOP. The main finding was that verbal learning was positively associated with global functioning, explaining 20% of the variance in the level of functioning, and that this association was significantly mediated by negative and disorganized symptoms. Our finding showing a significant association between verbal learning and global functioning is in accordance with two previous studies of patients with EOS [19, 29]. Our results are also in accordance with two previous studies including adult schizophrenia patients, showing that negative symptoms mediated the relationship between neurocognitive performance and global functioning, while positive symptoms did not [39, 40]. To the best of our knowledge, our study is the first to show that disorganized symptoms, as a separate construct from other positive symptoms, mediated the relationship between verbal learning and global functioning.

Verbal learning deficits are associated with earlier age of onset of psychosis [75] and transition to psychosis in high-risk individuals [76]. Deficits in this domain might be a predictor of, or contributing factor to, the clinical manifestation of negative and disorganized symptoms, which are considered to be a core feature of schizophrenia [77–79]. It has been suggested that the clinical heterogeneity in schizophrenia is a representation of different underlying mechanisms categorized by a positive and negative syndrome. The positive syndrome includes mainly positive symptoms (hallucinations and delusions) and better functional outcome, while the negative syndrome includes mainly negative and disorganized symptoms, cognitive deficits, and poorer functional outcome [35, 78–80]. Thus, the patients in our sample with verbal learning deficits, negative and disorganized symptoms could represent a negative subgroup with poorer global functioning [35, 78–80] and a more general language deficit, possibly due to a dysfunction in the language-processing networks [81].

Contrary to our hypothesis, we did not find significant associations between global functioning and other neurocognitive domains or global cognition, which have been found in previous studies of EOS and in adult schizophrenia [19, 29, 49, 82]. One reason for the different results between our study and the two previous studies of adolescents [19, 29] may be that in the two previous studies, functional outcome was measured only at follow-up and compared with baseline neurocognitive functioning, while we only applied baseline data. Comparing our results to previous studies of adult patients [49, 82], the divergence in findings in our study might be due to different factors influencing neurocognitive functioning in adolescence as compared to in adulthood. For example, more supportive factors in the adolescents’ rearing environment could help them to maintain their level of functioning. Moreover, different measures of neurocognition and functional outcome were applied in our study and the previous studies. However, our analyses showed that verbal learning and global functioning were more strongly associated with negative and disorganized symptoms than other symptoms. These findings are in accordance with a previous study showing that negative and disorganized symptoms showed stronger associations with neurocognitive deficits, than positive symptoms [35]. Moreover, negative symptoms have also been more strongly associated with impaired functional outcome in EOP [36] and adult schizophrenia [37, 38], compared to positive symptoms.

### Strengths and limitations

The main strength of this study is the large and well-characterized cohort of adolescent EOP patients. However, the study has several limitations. First, the study was naturalistic and cross-sectional, and no empirical assumptions of causality could be made, only investigations of statistical associations. We assume that baseline deficits in verbal learning and the presence of negative and disorganized symptoms will affect long-term global functioning in adolescents with EOP, but this must be investigated in a follow-up study. Second, global functioning was assessed using two different scales. Although the scales were considered to be sufficiently similar to merge and we controlled for cohort effects in the analyses, we cannot be certain that this did not bias the ratings of global functioning since no estimate of agreement between the two scales have been provided. Moreover, the ratings of functioning were based on the subjective impression of the interviewer. The interviewers were trained in scoring of CGAS and GAF, but no inter-rater reliability testing or observation of actual functioning was completed. Third, when performing separate analyses of the two cohorts, we found cohort differences in the regression and mediation analyses. Although cohort was added as a covariate in all the analyses, we cannot rule out that cohort differences may have biased the results. Fourth, it is unknown if medication effects have contributed to our findings as the combined cohort included medicated and unmedicated participants. Lastly, patient motivation was not assessed, although it has been shown to be related to cognitive performance in adult schizophrenia patients [83].

## Conclusion

Our results confirm that verbal learning is an important neurocognitive domain for global functioning and that negative and disorganized symptoms mediate this relationship, while reality-distorted positive symptoms do not. Hence, our results support the notion that disorganized symptoms should be considered as an independent construct separate from other positive symptoms [33, 34]. Clinical implications of the study are that assessments of adolescents with EOP should include measures of verbal learning, negative and disorganized symptoms. This could be of value for cognitive remediation programs and in planning of psychosocial and psychopharmacological interventions aiming to improve global functioning.

## Electronic supplementary material

Below is the link to the electronic supplementary material.

Supplementary file1 (DOCX 20 kb)
