# Supplementary material for: Negative and disorganized symptoms mediate the relationship between verbal learning and global functioning in adolescents with early-onset psychosis
Source: Eur Child Adolesc Psychiatry. 2020 Feb 8;29(12):1693–703. doi: 10.1007/s00787-020-01479-7 (PMC7641937; doi:10.1007/s00787-020-01479-7)
Supplement: Supplementary file 1 — Supplementary file1 (DOCX 20 kb) [file 787_2020_1479_MOESM1_ESM.docx]

**Separate statistical analyses for the two cohorts**

**The Early-Onset Study**

**Associations between neurocognitive performance, global functioning, and symptoms**

Using Bonferroni to correct for multiple testing, *P* ≤ .007 was considered significant (*P* = .05 / 7 neurocognitive domains). After correcting for multiple testing, Verbal learning was the only neurocognitive domain significantly associated with global functioning when controlling for age and sex (β = .50, *P* = .006, *R^2^* change = .21, model statistics (MS): *F*(3, 23) = 6.73, *P* = .002, *R^2^* = .47). For the other domains and the global cognition score, no associations reached statistical significance (Speed of processing: β = .34, *P* = .07, *R^2^* change = .11, MS: *F*(3, 22) = 3.89, *P* = .02, *R^2^* = .35; Attention/vigilance: β = .24, *P* = .23, *R^2^* change = .05, MS: *F*(3, 23) = 3.32, *P* = .04, *R^2^* = .30; Working memory: β = .17, *P* = .34, *R^2^* change = .03, MS: *F*(3, 22) = 3.50, *P* = .03, *R^2^* = .32; Visual learning: β = .05, *P* = .80, *R^2^* change = .00. MS: *F*(3, 23) = 2.66, *P* = .07, *R^2^* = .26; Reasoning and problem solving: β = .16, *P* = .39, *R^2^* change = .02, MS: *F*(3, 23) = 2.97, *P* = .05, *R^2^* = .28; Global cognition: β = .31, *P* = .09, *R^2^* change = .09, MS: *F*(3, 21) = 4.10, *P* = .02, *R^2^* = .37).

Because Verbal learning was the only neurocognitive domain significantly associated with global functioning, it was the only domain included in the subsequent analyses between neurocognition and symptoms. Bonferroni was applied to correct for multiple testing, in which *P* ≤ .01 was considered significant (*P* = .05 / 5 symptom factors). No symptom factors were significantly associated with Verbal learning, controlling for age and sex (Positive: β = -.08, *P* = .70, *R^2^* change = .01, MS: *F*(3, 23) = 1.29, *P* = .30, *R^2^* = .14; Negative: β = -.55, *P* = .04, *R^2^* change = .15, MS: *F*(3, 23) = 3.17, *P* = .04, *R^2^* = .29; Disorganized/concrete: β = -.42, *P* = .03, *R^2^* change = .16, MS: *F*(3, 23) = 3.33, *P* = .04, *R^2^* = .30; Excited: β = -.34, *P* = .08, *R^2^* change = .11, MS: *F*(3, 23) = 2.51, *P* = .08, *R^2^* = .25; Depressed: β = .03, *P* = .89, *R^2^* change = .00, MS: *F*(3, 23) = 1.24, *P* = .32, *R^2^* = .14).

We investigating associations between symptom factors and global functioning, controlling for age and sex. Bonferroni was applied to correct for multiple testing, in which *P* ≤ .01 was considered significant (*P* = .05 / 5 symptom factors). The Disorganized/concrete (β = -.47, *P* = .008, *R^2^* change = .20, MS: *F*(3, 23) = 6.44, *P* = .003, *R^2^* = .46) and Excited (β = -.50, *P* = .003, *R^2^* change = .24, MS: *F*(3, 23) = 7.43, *P* = .001, *R^2^* = .49)

symptom factors were significantly negatively associated with poorer global functioning. The Positive (β = -.25, *P* = .16, *R^2^* change = .06, MS: *F*(3, 23) = 3.56, *P* = .03, *R^2^* = .32), Negative (β = -.41, *P* = .10, *R^2^* change = .08, MS: *F*(3, 23) = 3.94, *P* = .02, *R^2^* = .34) and Depressed (β = -.37, *P* = .04, *R^2^* change = .13, MS: *F*(3, 23) = 4.80, *P* = .01, *R^2^* = .39) symptom factors did not reach statistical significance.

**Symptoms as mediators of the association between neurocognitive performance and global functioning**

Verbal learning was the only neurocognitive domain significantly associated with global functioning and thus the only domain included in the mediation analyses for testing the indirect effects. The Excited (point estimate = 2.13, BC 95% CI = .03, 6.86) symptom factor significantly mediated (CI not including the null value) the relationship between Verbal learning and global functioning, controlling for age and sex. None of the other symptom factors reached statistical significance (Positive: point estimate = .28, BC 95% CI = -1.18, 3.17; Negative: point estimate = .80, BC 95% CI = -.76, 3.89; Disorganized/concrete: point estimate = 2.17, BC 95% CI = -.01, 8.25; Depressed: point estimate = -.17, BC 95% CI = -2.89, 2.61).

**Youth-TOP**

**Associations between neurocognitive performance, global functioning, and symptoms**

Using Bonferroni to correct for multiple testing, *P* ≤ .007 was considered significant (*P* = .05 / 7 neurocognitive domains). After correcting for multiple testing, no associations between neurocognition and global functioning reached statistical significance, when controlling for age and sex (Speed of processing: β = .15, *P* = .43, *R^2^* change = .02, MS: *F*(3, 29) = .48, *P* = .70, *R^2^* = .05; Attention/vigilance: β = -.03, *P* = .88, *R^2^* change = .00, MS: *F*(3, 25) = .21, *P* = .89, *R^2^* = .03; Working memory: β = -.10, *P* = .59, *R^2^* change = .01, MS: *F*(3, 30) = .33, *P* = .80, *R^2^* = .03; Verbal learning: β = .46, *P* = .02, *R^2^* change = .18, MS: *F*(3, 29) = 2.46, *P* = .08, *R^2^* = .20; Visual learning: β = .36, *P* = .05, *R^2^* change = .12, MS: *F*(3, 30) = 1.68, *P* = .19, *R^2^* = .14; Reasoning and problem solving: β = -.02, *P* = .91, *R^2^* change = .00, MS: *F*(3, 30) = .23, *P* = .87, *R^2^* = .02; Global cognition: β = .04, *P* = .88, *R^2^* change = .00, MS: *F*(3, 23) = .19, *P* = .90, *R^2^* = .02). Although no associations between the neurocognitive domains and global functioning reached statistical significance, we decided to perform subsequent separate analyses in the Youth-TOP with the Verbal learning domain, as this domain is included in the main analyses in the combined cohort in the main text.

We investigated associations between the Verbal learning domain and symptoms, controlling for age and sex. Bonferroni was applied to correct for multiple testing, in which *P* ≤ .01 was considered significant (*P* = .05 / 5 symptom factors). No symptom factors were significantly associated with Verbal learning (Positive: β = -.09, *P* = .61, *R^2^* change = .01, MS: *F*(3, 29) = 1.67, *P* = .20, *R^2^* = .15; Negative (β = -.28, *P* = .11, *R^2^* change = .07, MS: *F*(3, 29) = 2.61, *P* = .07, *R^2^* = .21; Disorganized/concrete (β = -.39, *P* = .02, *R^2^* change = .14, MS: *F*(3, 29) = 3.78, *P* = .02, *R^2^* = .28; Excited: β = .08, *P* = .66, *R^2^* change = .01, MS: *F*(3, 29) = 1.64, *P* = .20, *R^2^* = .15; Depressed: β = .11, *P* = .54, *R^2^* change = .01, MS: *F*(3, 29) = 1.71, *P* = .19, *R^2^* = .15).

We investigating associations between symptom factors and global functioning, controlling for age and sex. Bonferroni was applied to correct for multiple testing, in which *P* ≤ .01 was considered significant (*P* = .05 / 5 symptom factors). The Negative (β = -.57, *P* = .001, *R^2^* change = .31, MS: *F*(3, 30) = 5.01, *P* = .006, *R^2^* = .33) symptom factor was significantly negatively associated with poorer global functioning. The associations between the other symptom factors and global functioning did not reach statistical significance (Positive: β = -.24, *P* = .20, *R^2^* change = .05, MS: *F*(3, 30) = .82, *P* = .49, *R^2^* = .08; Disorganized/concrete: β = -.37, *P* = .04, *R^2^* change = .13, MS: *F*(3, 30) = 1.77, *P* = .18, *R^2^* = .15; Excited: β = .05, *P* = .81, *R^2^* change = .00, MS: *F*(3, 30) = .25, *P* = .86, *R^2^* = .02; Depressed: β = .12, *P* = .55, *R^2^* change = .01, MS: *F*(3, 30) = .35, *P* = .79, *R^2^* = .03).

**Symptoms as mediators of the association between neurocognitive performance and global functioning**

Because Verbal learning was included in the mediation analyses in the combined cohort in the main text, we performed mediation analyses for testing the indirect effects of this domain in the Youth-TOP. None of the symptom factors significantly mediated the relationship between Verbal learning and global functioning, controlling for age and sex (Positive: point estimate = .22, BC 95% CI = -.71, 3.62; Negative: point estimate = .1.70, BC 95% CI = -.35, 5.53; Disorganized/concrete: point estimate = 1.13, BC 95% CI = -.34, 6.35; Excited (point estimate = .01, BC 95% CI = -.85, 1.25; Depressed: point estimate = -.08, BC 95% CI = -.62, 1.55).
